# Macrophages and fibrosis: how resident and infiltrating mononuclear phagocytes account for organ injury, regeneration or atrophy

**DOI:** 10.3389/fimmu.2023.1194988

**Published:** 2023-10-06

**Authors:** Hao Long, Julia Lichtnekert, Joachim Andrassy, Barbara U. Schraml, Paola Romagnani, Hans-Joachim Anders

**Affiliations:** ^1^ Division of Nephrology, Department of Medicine IV, University Hospital, Ludwig-Maximilians-University (LMU), Munich, Germany; ^2^ Department of Urology, The Affiliated Hospital of Southwest Medical University, Luzhou, China; ^3^ Sichuan Clinical Research Center for Nephropathy, Luzhou, China; ^4^ Department of General, Visceral and Transplant Surgery, University Hospital of Ludwig-Maximilians-University (LMU) Munich, Munich, Germany; ^5^ Institute for Cardiovascular Physiology and Pathophysiology, Biomedical Center, Ludwig-Maximilians-University (LMU), Munich, Germany; ^6^ Walter-Brendel-Centre of Experimental Medicine, University Hospital, Ludwig-Maximilians-University (LMU), Munich, Germany; ^7^ Department of Biomedical, Experimental and Clinical Sciences “Mario Serio”, University of Firenze, Nephrology and Dialysis Unit, Meyer Children’s Hospital, Firenze, Italy

**Keywords:** dendritic cells, macrophages, inflammation, necrosis, fibrosis, polyploidy

## Abstract

Mononuclear phagocytes (MP), i.e., monocytes, macrophages, and dendritic cells (DCs), are essential for immune homeostasis via their capacities to clear pathogens, pathogen components, and non-infectious particles. However, tissue injury-related changes in local microenvironments activate resident and infiltrating MP towards pro-inflammatory phenotypes that contribute to inflammation by secreting additional inflammatory mediators. Efficient control of injurious factors leads to a switch of MP phenotype, which changes the microenvironment towards the resolution of inflammation. In the same way, MP endorses adaptive structural responses leading to either compensatory hypertrophy of surviving cells, tissue regeneration from local tissue progenitor cells, or tissue fibrosis and atrophy. Under certain circumstances, MP contribute to the reversal of tissue fibrosis by clearance of the extracellular matrix. Here we give an update on the tissue microenvironment-related factors that, upon tissue injury, instruct resident and infiltrating MP how to support host defense and recover tissue function and integrity. We propose that MP are not intrinsically active drivers of organ injury and dysfunction but dynamic amplifiers (and biomarkers) of specific tissue microenvironments that vary across spatial and temporal contexts. Therefore, MP receptors are frequently redundant and suboptimal targets for specific therapeutic interventions compared to molecular targets upstream in adaptive humoral or cellular stress response pathways that influence tissue milieus at a contextual level.

## 1 Introduction

Tissue fibrosis is defined by an excess of extracellular matrix (ECM) and characterized by tissue stiffness (sclerosis), which can impair the function of elastic organs such as the heart, lungs, or skin (1). In addition, excess ECM can impair transepithelial transport functions, e.g., in the lungs (air-blood-air) and the kidneys (urine-blood-urine) (2). Apart from progressive scleroderma, where autoimmunity directly triggers fibrosis in otherwise healthy organs, in most cases, fibrosis does not spread into healthy tissue, e.g., in dermal wound healing (3). Generally, fibrosis instead serves as a marker for adaptive mechanisms responding to focal or diffuse parenchymal injury. For example, in the heart, liver, and kidney, interstitial fibrosis is a typical result of ischemic or toxic parenchymal damage leading to compensatory parenchymal cell hypertrophy, a process involving cell cycle entry, polyploidization, and a hypersecretory cell state involving the secretion of pro-fibrotic mediators (4). In this context, tissue-resident or infiltrating MP amplify the pro-fibrotic microenvironment, fibroblasts, and downstream ECM producers (5, 6). Here we focus on the role of MP in tissue injury and repair to understand better their role in fibrogenesis. We briefly summarize the spectrum of tissue-resident and infiltrating MP and the recent progress in their role in the different phases of injury, repair, and progressive and reversible fibrosis.

## 2 Development and definition of mononuclear phagocytes

**Figure 1:**
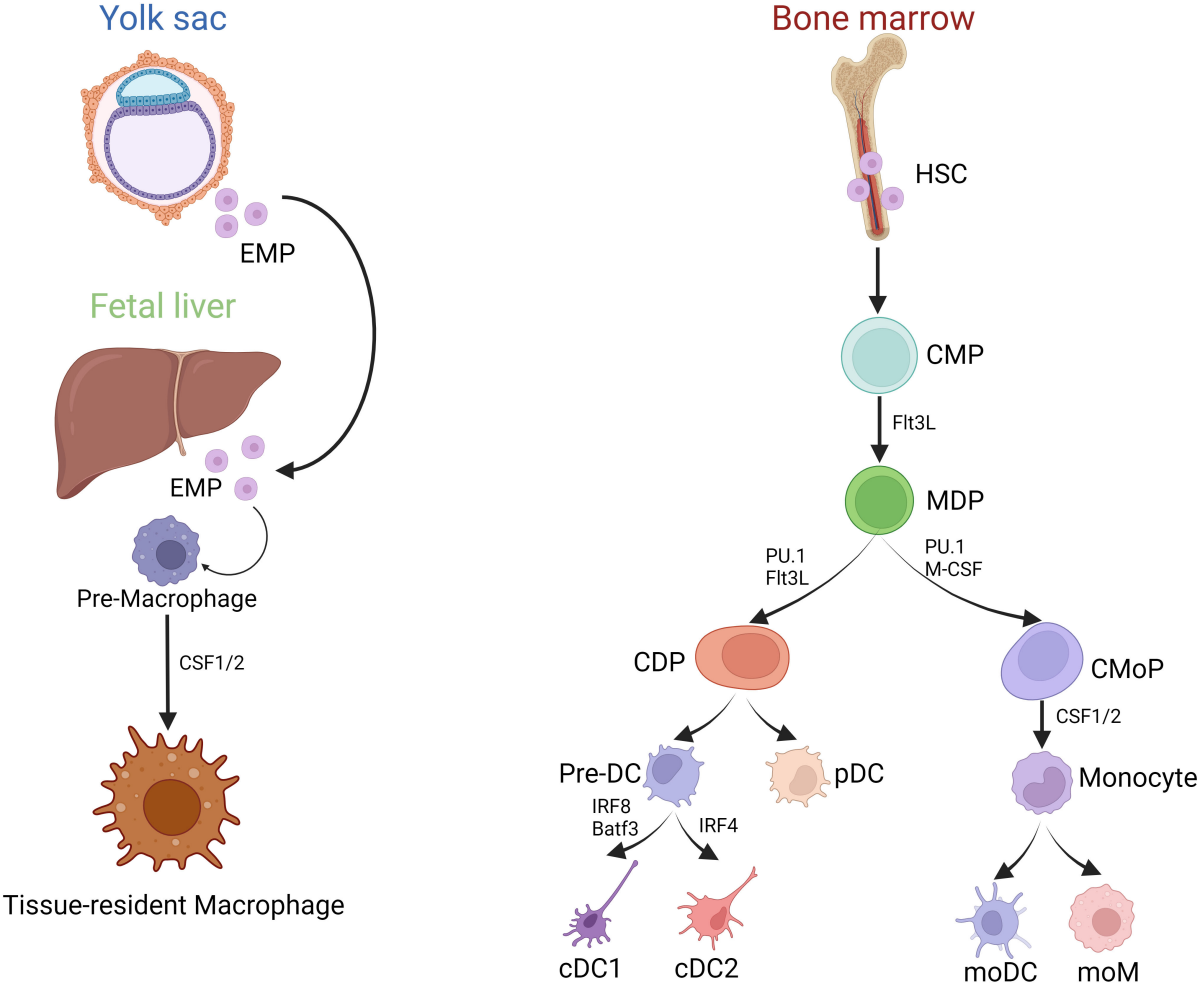
Development of MP. (Created with BioRender.com) Development of MP mainly includes two sources: (1) Yolk sac and Fetal liver erythrocyte-myeloid progenitor (EMP)-derived way; (2) Bone marrow HSC derived way. Tissue-resident macrophages primarily originate from embryonic yolk sac EMPs and fetal liver EMPs, whereas bone marrow-derived monocytes enter the tissues during inflammatory conditions as infiltrating cells, and then differentiate into moDC and moM. However, bone marrow HSCs can evolve into CMPs, and part of them can differentiate into MDPs under the stimulation of Flt3L. MDPs are directed precursors of CDPs and CMoPs. After evolving, CDPs can produce each subgroup of the DCs series, including precursor DC (pre-DC) and pDC. IRF8 is required for the lineage development of cDC1s, helping to define distinct cDC1 subsets. IRF4, a nuclear factor interacting with PU.1, is essential for many aspects of cDC2 function.

MP comprising macrophages, monocytes, and DCs can be found in all lymphoid and non-lymphoid tissues (7). Macrophages develop from two distinct haematopoetic lineages and can therefore be divided in fetal-derived and monocyte derived macrophages. Fetal-derived macrophages arise from erythro-myeloid progenitors from the yolk sac and colonize the fetal liver before birth. Some of these macrophages persist into adulthood as resident cells in various tissues and are able to self-renew (**Figure 1**) (8). The expression of macrophage colony-stimulating factor 1 receptor (CSF-1R) plays a pivotal role in regulating the development of macrophages. In mice lacking CSF-1 tissue-resident macrophages in most organs are absent (9).

In contrast, monocytes and DCs derive from bone marrow progenitors. In the bone marrow, hematopoietic stem cells (HSCs) can differentiate into common myeloid progenitor cells (CMPs). When stimulated by FMS-like tyrosine kinase 3 ligand (Flt3L), some of these CMPs can further develop into macrophage and dendritic cell progenitor cells (MDPs). MDPs represent a specific precursor for both common dendritic cell progenitor cells (CDPs) and monocyte progenitor cells (CMoPs). CMoPs can differentiate into monocytes under the stimulation of CSF 1/2. During inflammation, macrophages can derive from monocytes, called monocyte-derived macrophages (moM) (**Figure 1**). CDPs, derived from MDPs, can be further categorized into plasmacytoid dendritic cells (pDCs) and conventional dendritic cells (cDCs). pDCs are particularly known for their capacity to secrete alpha interferons (IFN), while cDCs are considered highly effective in activating T-cell responses (7). cDCs further divide into IRF8 and Batf3-dependent type I conventional dendritic cells (cDC1s) that excel at cross-presentation and IRF4-expressing type II conventional dendritic cells (cDC2s), best known for their ability to drive T helper (Th) cell differentiation (10, 11). cDC2s are heterogeneous, with subpopulations with non-redundant functions in Th17 and Th2 responses regulated by NOTCH2 and KLF4, respectively (12). In addition, the development of cDCs and pDCs relies mainly on the hematopoietic cytokine Flt3L.

Historically, macrophages were defined by positivity for F4/80 and DCs by positivity for CD11c and major histocompatibility complex (MHC) II. However, macrophages, monocytes, and DCs may overlap in phenotype and function (7, 13), and this is particularly prominent for example in the kidney, which is why macrophages and DCs in the kidney are referred to as renal MP (14). In addition, surface marker expression profiles are dynamic and depend on cell maturation and activation status (15), which makes it difficult to attribute specific functions to specific subtypes of cells.

Flt3L stimulates the differentiation of both populations of dendritic cells *in vitro*, providing an accurate reflection of the physiological process. On the other hand, culturing bone marrow (BM) cells in the presence of granulocyte-macrophage colony-stimulating factor (GM-CSF) results in DCs resembling those derived from monocytes (moDC). However, when BM cells are cultured with a combination of GM-CSF and Flt3L, generating a significant quantity of CD103^+^ DCs is possible, which is a promising target for tolerance induction or vaccination (16).

A way of classifying macrophages has been assessed by *in vitro* assays where, under the influence of various cytokines representing distinct tissue microenvironments, macrophages turn into individual phenotypes with specific secretome and functional capacities (**Table 1**). Nevertheless, the spectrum of different techniques used to describe MP to distinct cell populations is one of the reasons for some uncertainty in the nomenclature (17).

**Table 1:** Types of macrophages and their related phenotypes.

M0	M1	M2a	M2b	M2c
Tissue-resident macrophages, highly plastic, multifunctional cells	Classic activation phenotype, Pro-inflammatory macrophages, Support the development of Th1	Alternative activation phenotype and anti-inflammatory/pro-fibrotic macrophages, Support the development of Th2		
	Pro-inflammatory and host defense phenotype	Anti-inflammatory Phenotype, promote Wound healing and tissue fibrosis	Anti-inflammatory phenotype, immunoregulation	Pro-regenerative and healing phenotype
	Induced by LPS, IFN-γ	Mainly induced by IL-4	Induced by immune compound and IL-1R and/or TLR ligands	Induced by IL-10, TGF-β, glucocorticoids
	Secrete TNF-α, IL-1, IL-6, IL-12, IL-23, iNOS, MMP12, MINCLE	Secrete significant amounts of IL-10 and IL-1R antagonists	Contribute to Th2-like activation, produce IL-10	Secrete TGF-β, IL-10, ARG1
	Upregulate MHC-II, CD16, CD32, CD80, CD86	Down-regulate IL-1β, IL-12, NO	Downregulate TNF-α, IL-1β, IL-12, IL-6	Downregulate TNF-α, IL-1β, IL-12, IL-6

Th1(T helper cells type 1), LPS (Lopopolysaccharides), IFN-γ (Interferon γ), TNF-α (Tumor Necrosis Factor α), IL (Interleukin), iNOS (Inducible Nitric Oxide Synthase), MMP12(Matrix Metalloproteinase 12), MINCLE (Macrophage-inducible C-type Lectin), MHC-II (Major Histocompatibility Complex Class II), Th2(T helper cells type 2), NO (Nitrogen Oxide), IL-1R (Interleukin 1 Receptor), TLR (Toll-like Receptor), TGF-β (Transforming Growth Factor β), ARG1 (Arginase 1).

Refined lineage-tracing technologies of macrophages, monocytes, and DCs have facilitated the distinction of these cells (13, 15, 18–20). Therefore, a classification of MP subtypes based on cell origin has been suggested (21). Such a definition has limitations and may not be tenable in the dynamics of acute disorders with changing microenvironments when cell types exhibit phenotypic plasticity, and lineage tracing-related definitions are less valuable. However, it has become clear that MP subtypes have specific functions in pathology (22, 23). Defining cell types by lineage-specific transcription factors is a powerful tool to establish the functions of MP in immunity. Recently, lineage-specific transcription factors have been used to determine the functions of different MP lineages, such as MAFB for macrophages and ZBTB46 for DCs (24). Likewise, IRF4, a nuclear factor that interacts with PU.1, is essential for the many aspects of cDC2s function but also mediates macrophage polarization to the M2 phenotype (21). However, such models have limitations, e.g., expression by other immune cell lineages (25) (**Table 2**).

**Table 2:** Different lineages of mouse MP.

MP	Subsets	Surface Markers	Transcription factors	Functions
Monocytes	Ly6C high inflammatory	Ly6C high, CCR2 high	KLF4	Differentiate into DCs and tissue macrophages during inflammation
Macrophages	Ly6C low patrolling	Ly6C low, CCR2 low, Cx3CR1	MAFB	Endothelial integrality
	Tissue-specific	F4/80, MERTK, CD64, CD11b	MAFB	Tissue-specific: Lungs (Alveolar macrophages), Bone (osteoclasts), Liver (Kupffer cells), etc.
Dendritic cells	pDCs	SIGLEC H, BST2, CD123, BDCA2, AXL, CD45RA, CD33	TCF4(E2-2), ZEB2	Production of type I IFNs
	cDC1s	XCR1, CD103, Clec9a, CD11c, MHC-II, CD205	ZBTB46, IRF8	Th1 and CTL immune, cross- presentation, IL-12 production
	cDC2s	CD11b, CD11c, SIRP-α, MHC-ii, CD205	ZBTB46, IRF4, NOTCH2, KLF4	Th2 and Th17 immune, IL-23 and IL-6 production

MP (Mononuclear Phagocytes), pDCs (plasmacytoid dendritic cells), cDC1s (Type I conventional dendritic cells), cDC2s (Type II conventional dendritic cells), CCR2 (C-C motif Chemokine Receptor 2), CX3CR1 (C-X3-C motif Chemokine Receptor 1), MERTK (MER Proto Oncogene, Tyrosine Kinase), SIGLEC-H (Sialic acid binding Ig-like Lectin H), BST2 (Bone Marrow Stromal Cell Antigen 2), BDCA2 (Blood Dendritic Cell Antigen 2), XCR1 (X-C motif Chemokine Receptor 1), Clec9a (C-type Lectin Domain Containing 9A), MHC-II (Major Histocompatibility Complex Class II), SIRP-α (Signal Regulatory Protein α), KLF4 (Kruppel-like factor 4), MAFB (MAF BZIP Transcription Factor B), TCF4 (Transcription factor 4), ZEB2 (Zinc Finger E-Box Binding Homeobox 2), ZBTB46 (Zinc finger and BTB domain containing 46), IRF8 (Interferon Regulatory Factor 8), IRF4 (Interferon Regulatory Factor 4), NOTCH2 (Neurogenic locus notch homolog protein 2), IFNs (Interferons), Th1(T helper cells type 1), CTL (Cytotoxic T cell), IL (Interleukin), Th2(T helper cells type 2), Th17(T helper cells type 17).

## 3 Mononuclear phagocytes in tissue injury and repair

These highly phagocytic cells sense their environment for signs of damage or pathogens and initiate immune responses. MP are present in different organs, e.g. in the bone, where the turnover process requires osteoclasts, while in the liver, Kupffer cells are in charge of eliminating pathogens and pathogen components from the blood in the portal vein, clearing old red blood cells, and recovering iron ions (26). MP reside in different compartments in the same organ. For example, macrophages located in the alveolar space are called alveolar macrophages, while macrophages found in interstitial compartments are referred to as interstitial macrophages (27). Here, we describe the different microenvironments along tissue injury and repair and discuss the respective contribution of MP in these contexts.

### 3.1 Tissue injury and necroinflammation

Invasion of pathogens, exposure to toxins, metabolic stress, ischemia, trauma, or the presence of malignant cells are triggers of cell injury and the release of Danger-Associated Molecular Patterns (DAMPs) or Pathogen-Associated Molecular Patterns (PAMPs), respectively. These molecules trigger immune responses and hence promote further tissue damage. PAMPs occur on the surface of pathogens, including lipopolysaccharide (LPS) and bacterial or viral nucleic acids. DAMPs are released from damaged or dying cells, including intracellular molecules, such as S100A9 proteins, HMGB1, uric acid, and histones. PAMPs and DAMPs have the identical ability to activate pattern recognition receptors (PRRs) of the innate immune system, such as Toll-like receptors (TLRs), NOD-like receptors (NLRs), C-type lectin receptors (CLRs) or inflammasomes to secrete multiple pro-inflammatory mediators that induce local inflammation (9, 28–30). Parenchymal cells and resident MP are the first cell types that sense danger and initiate evolutionarily conserved defense mechanisms that create a barrier to intruding pathogens. Histamine and other vasoactive mediators induce endothelial leakage, allowing serum components such as immunoglobulins and other opsonin to reach the injury site. Local chemokine release and upregulation of adhesion molecules on the luminal endothelial surface facilitate the transmigration of first neutrophils and subsequently CC-chemokine receptor 2^+^ (CCR2^+^) monocytes to limit pathogen spreading by direct killing and phagocytic clearance (31, 32). Upon arrival, such monocytes encounter a microenvironment characterized by DAMPs (in case of infection also PAMPs), chemokines such as C-chemokine ligand-2 (CCL2), chemokine fractalkine (CX3CL1), etc., which specifically attract monocytes. Once at the injury site, monocytes can serve as effectors or further differentiate into moM or moDC (33, 34). Meanwhile, pro-inflammatory cytokines and lipid mediators induce a pro-inflammatory macrophage phenotype, which is difficult to distinguish from activated resident macrophage populations by surface markers. Such pro-inflammatory macrophage phenotypes share similarities with cultured macrophages stimulated with IFN-γ and LPS, referred to as the M1 phenotype which supports the development of Th1. M1 macrophages are characterized by activation markers on the cell surface and molecules involved in antigen presentation, including MHC-II, CD16, CD32, CD80, and CD86 (35–38). Meanwhile, M1 macrophages enhance the inflammatory microenvironment by producing pro-inflammatory mediators, such as TNF-α, IL-1, IL-6, IL-12, IL-23, and other molecules such as inducible nitric oxide synthase (iNOS), matrix metalloproteinase 12 (MMP-12), and macrophage-induced C-type lectin (MINCLE) (**Table 1**). This contribution to the inflammatory milieu leads to the initiation of various forms of regulated cell death, like necrosis, apoptosis, and pyroptosis. Moreover, when cells undergo necrosis, they release additional DAMPs, further activating neighboring MP. The auto-amplification loop is called necroinflammation (39, 40).

Furthermore, different subsets of DCs accelerate tissue inflammation in a complementary manner. pDCs release large amounts of type I IFNs essential for antiviral immune defense (41, 42). Tissue-resident cDC1s and cDC2s detect immunogenic substances and further recognize and release pro-inflammatory mediators such as CXCL2, IL-12, and IL-6 (14). Significantly CXCL2 changes the microenvironment by recruiting neutrophils, which promote necroinflammation (43). In addition to cytokine production, cDC1s, and cDC2s enter regional lymph nodes to initiate adaptive immune responses. Among them, a unique feature of cDC1s is the ability to cross-present antigens to CD8^+^ T cells, whereas cDC2s cross-present antigens to CD4^+^ T cells (44, 45).

Recently, the multifaceted role of the adaptive immune system in the pro-inflammatory environment of the damaged heart after acute myocardial infarction has been elucidated (46). Under the stress of chronic overload, DCs accumulate potent γ-ketoaldehydes, which activate the pro-inflammatory program of reactive oxygen species (ROS) and the secretion of IL-1β, IL-6, and IL-23 (47). DCs can also increase the expression of T-cell co-stimulatory proteins. In a hypertensive state, heart DCs promote the proliferation of T cells, particularly CD8^+^ T cells, and contribute to their polarization towards a pro-inflammatory phenotype (47–49). So, DCs play a pro-inflammatory role in cardiac injury by sustaining oxidative stress, releasing pro-inflammatory factors, and activating T cells.

### 3.2 Resolution of inflammation

*In vitro* studies dissect three subsets of M2 macrophages based on their phenotypes and functions (**Table 1**). M2a macrophages respond to IL-4 and IL-13 and show a predominantly anti-inflammatory phenotype. They secrete high levels of IL-10 and IL-1 receptor antagonists, and growth factors that aid tissue healing by stabilizing angiogenesis, such as platelet-derived growth factor (PDGF), transforming growth factor β (TGF-β), etc. which help to suppress the inflammatory response (50, 51). Additionally, M2a macrophages can induce an anti-inflammatory Th2-like immune response, promote wound healing, and contribute to tissue fibrosis. On the other hand, M2b macrophages can participate in immunoregulation and contribute to Th2-like activation induced by immune complexes and TLR and/or IL-1R ligands. They produce IL-10, which further contributes to the anti-inflammatory response. M2c macrophages are activated by various factors such as IL-10, TGF-β, and glucocorticoids. These stimuli induce a specific phenotype in M2c macrophages characterized by their ability to promote tissue repair and inhibit tissue inflammation (52–54).. Control of inflammation is essential to limit immunopathology. Therefore, the activation of pro-inflammatory signaling pathways precedes their deactivation via the subsequent induction of anti-inflammatory signaling pathways. Well-known phenomena of endotoxin tolerance or ischemic preconditioning represent this process. For example, macrophages participate in efferocytosis, a process in which they engulf apoptotic neutrophils (31, 55). Efferocytosis initiates the resolution of inflammation, which prevents further neutrophil recruitment and eliminates neutrophils silently before they may undergo secondary necrosis (56, 57). Efferocytosis induces a shift to an “alternately activated” macrophage phenotype, also known as the M2 type (**Table 1**). The interaction between M2 macrophages and the adaptive immune system is critical for resolving inflammatory responses in multiple tissues, especially with Th2 cells (58) and regulatory T cells (Tregs) (59–61). M2 macrophages stimulate epicardial progenitor cell proliferation (62) and promote the secretion of ECM molecules and tissue remodeling (63). In contrast to M1 macrophages, M2 macrophages express the enzyme arginase 1 (ARG1) constitutively. ARG1 is responsible for hydrolyzing L-arginine into L-ornithine, a crucial precursor for producing polyamines necessary for cell survival. Additionally, L-ornithine can generate proline and hydroxyproline, essential amino acids required for collagen synthesis. Collagen is vital in maintaining the structural integrity of non-injured tissue parenchyma (64, 65).

DCs exhibit their contributions to the innate and adaptive immune systems as gatekeepers for the induction of adaptive immunity. Harmless foreign proteins circulating in the bloodstream get filtered in the kidney, transported across proximal tubular epithelial cells, and can reach the kidney lymph nodes independent of DC uptake and processing. Once in the lymph node, local tolerogenic DCs process these proteins. This mechanism helps preventing immune responses against harmless foreign or self-proteins in the serum (66). Batf3^+^ DCs in the kidney lymph nodes present filtered antigens along with programmed cell death ligand 1(PD-L1) to cytotoxic T lymphocytes (CTLs), resulting in cross-tolerance development (67).

### 3.3 Tissue adaptation to injury: hypertrophy, regeneration, fibrosis

#### 3.3.1 Hypertrophy

**Figure 2:**
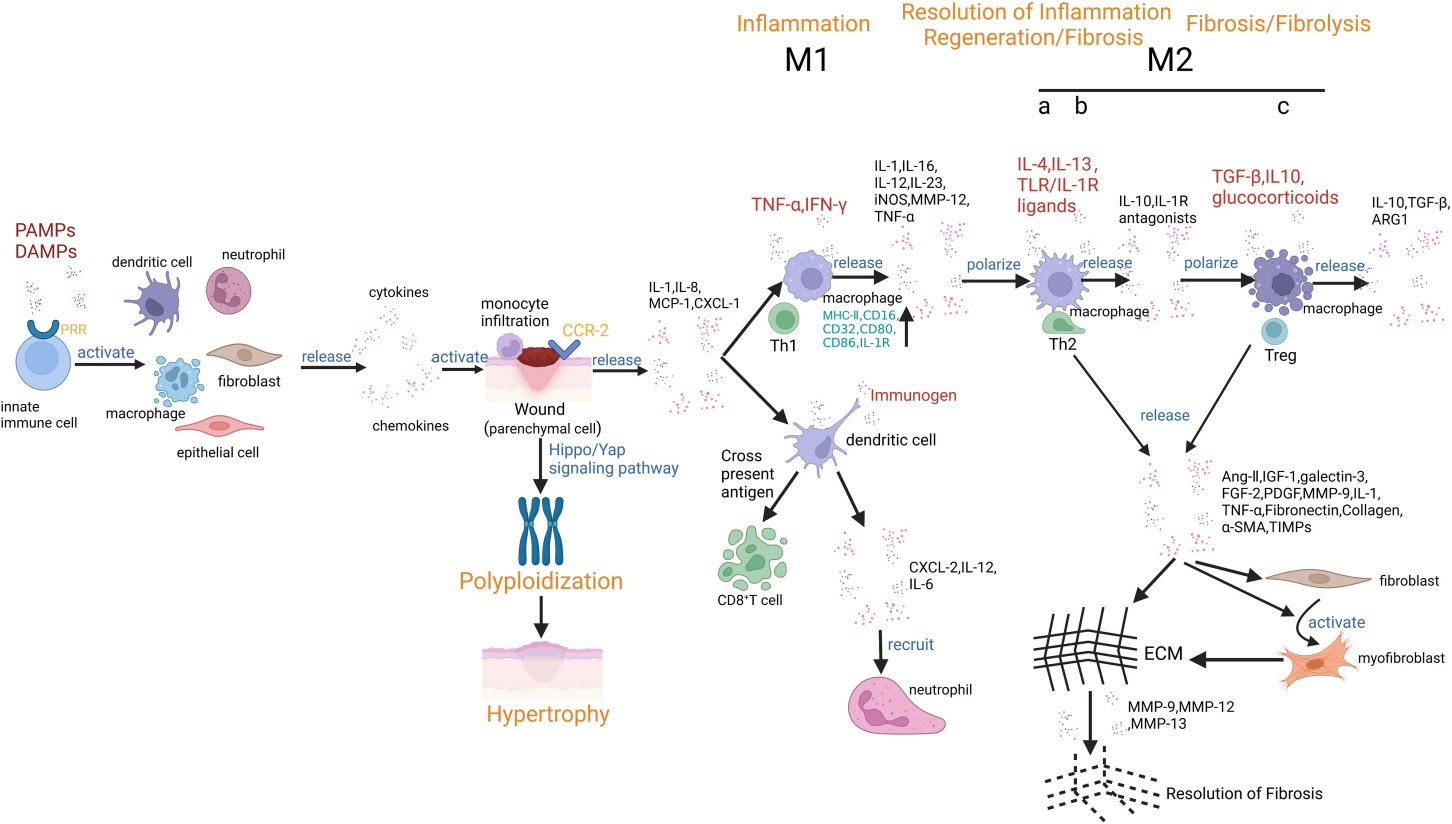
Mononuclear phagocytes and tissue fibrosis (Created with BioRender.com). The PRRs on innate immune cells recognize DAMPs or PAMPs and activate TRMs, neutrophils, DCs, fibroblasts, and endothelial cells, which release various pro-inflammatory chemokines and cytokines. Monocytes are abundantly recruited from the blood to sites of inflammation, secrete pro-inflammatory cytokines and chemokines, and differentiate into moM or moDC. Meanwhile, following injury, surviving differentiated parenchymal cells can increase their functional capacity by undergoing hypertrophy through polyploidization, all while maintaining their functional performance. One of the regulators of polyploidization is the Hippo/Yap signaling pathway. During tissue injury and early stages of inflammation, macrophages initially assume the M1 type. M1 macrophages are characterized by their role in host defense and production of pro-inflammatory cytokines. Meanwhile, distinct DC subsets promote tissue inflammation in a complementary manner. cDC1s and cDC2s are tissue-resident and recognize, activate, and release cytokines upon detection of immunogenic substances. The resulting changes in the microenvironment promote the recruitment of neutrophils. In addition, a unique feature of cDC1s is the ability to cross-present antigens to CD8^+^ T cells. Once the acute inflammatory phase is resolved, there is a shift in the predominant macrophage population towards the M2 type. M2 macrophages are characterized by the secretion of anti-inflammatory mediators and growth factors that aid tissue healing by stabilizing angiogenesis. It stimulates and promotes ECM assembly and remodeling. M2 macrophages are known to secrete numerous pro-fibrotic factors that contribute to the proliferation of fibroblasts, activation, and survival of myofibroblasts, as well as the excessive production of ECM. After removing the injury stimulus, macrophages will transition from a phenotype driven by the uptake of cellular debris to an anti-fibrotic phenotype and secrete multiple fibrinolytic MMPs, which enhance the degradation of fibrotic ECM.

The tissue response to injury differs across cell lineages and stages of differentiation. For example, upon injury and loss of organ function, surviving differentiated parenchymal cells respond with polyploidization to support organ function recovery by undergoing cell hypertrophy (**Figure 2**) (4). The Hippo/Yes-associated protein (Yap) signaling pathway is the primary regulator of polyploidization. Yap accelerates the growth of polyploid cells by regulating Skp2, an E3 ligase that targets p27 for proteolytic degradation. This mechanism occurs in various types of parenchymal cells, such as polyploid differentiated hepatocytes that drive functional recovery by increasing the output of the metabolic function and hepatocyte progenitors that drive the restoration of liver mass through proliferation and differentiation. Similarly, in the early phase of heart injury, polyploidization of cardiomyocytes and regeneration driven by cardiomyocyte progenitors are critical for maintaining heart function and restoring tissue integrity. However, polyploidization can have negative consequences in the long term, as it is associated with scarring and chronic heart failure (4). Although it has been demonstrated that macrophages can promote hypertension-induced cardiac hypertrophy and failure through miR-155-dependent paracrine signaling, and the absence of miR-155 can reduce pressure overload-induced cardiac hypertrophy and inflammation, the role of MP in this context is still uncertain (68).

In the kidney, both podocytes and tubular cells exhibit similar responses to injury, which include undergoing polyploidization-induced hypertrophy and progenitor cell proliferation (4). After acute kidney injury (AKI), most tubular epithelial cells (TECs) undergo endocycle-mediated hypertrophy, which supports function but not tissue regeneration. These endocyclic TECs can indicate irreversible TEC loss and may be a prognostic indicator of the risk of developing chronic kidney injury (CKD). TEC progenitors have a limited clonal response, and targeting tubular Pax2^+^ progenitors has been considered a potential treatment strategy for AKI (69). A recent study by De Chiara and colleagues showed that Yap1-driven polyploidization of tubular cells is a compensatory mechanism to enhance residual kidney function and prevent premature death in kidney failure resulting from AKI. However, tubular cell polyploidy promotes tubular cell senescence, progressive interstitial fibrosis, and AKI-CKD transition. On the other hand, blocking Yap1-driven polyploidization at a later stage can prevent the development of CKD and improve the GFR-loss who survive the early injury phase (70). Polyploidization is the result of an alternative cell cycle process known as endoreplication. Endoreplication can lead to the formation of mononucleated polyploid cells through the endocycle or mono/multinucleated polyploid cells through endomitosis (71, 72).

#### 3.3.2 Regeneration

Immature tissue progenitors respond with cell proliferation and differentiation to replace lost cells, i.e., regeneration (**Figure 2**). Regeneration refers to the replacement and complete reconstruction of damaged or lost tissue structures through the proliferation of cells and tissues. Repair may restore part of the original structure, but reconstruction is incomplete and leads to structural remodeling (73). Injuries are categorized based on the ability of the tissue to regenerate. For example, skeletal muscle, epithelial tissues, liver, etc., can recover from mild injuries quickly after the inflammation subsides. When scars form, they can regress over a few weeks as myofibroblasts, ECM, and inflammatory macrophage infiltrate resolve, allowing regeneration of the parenchyma, resulting in the restoration of normal tissue (74–76). Immune mediators support this process. For example, IL-22 is a cytokine with evident pro-regenerative characteristics. In the colon, CD11c^+^ DCs secrete IL-22, which signals through the transducer and activator of the STAT3 pathway to promote re-epithelialization. Similarly, following ischemia, tubules release DAMPs that activate TLR4 on renal DCs, producing IL-22 to expedite tubule recovery. Additionally, M2 macrophages in healing kidneys secrete Wingless and Int (Wnt) ligands, including Wnt7b, which activate the Wnt signaling pathway and encourage tubule recovery, and this demonstrates how the immune systems’ resident and infiltrating cells play an active role in supporting the regeneration process (77).

#### 3.3.3 Fibrosis

Interstitial fibroblasts respond with increased secretion of ECM to stabilize the integrity of the remaining parenchyma, leading to tissue fibrosis (**Figure 2**). Fibrosis occurs for example in response to acute kidney injury and contributes to the transition to chronic kidney disease, and is commonly associated with repetitive injuries or chronic wounds with inadequate vascular supply. The persistence of damaging factors, such as hepatitis C in the liver, or a prolonged inflammatory response that is insufficient in replacing damaged cells, can lead to prolonged damage (78–80). When tissues cannot fully restore tissue architecture, scarring occurs. Severe injury implies scarring even in tissues with high regenerative capacity, such as skin (68). Tissues with limited regenerative potential, such as the brain and heart, undergo rapid healing via the formation of a scar, but this comes at the cost of organ function (81). Over several months, the scar undergoes a maturation process, forming clusters of ECM, myofibroblasts, and macrophages (82). The canonical Wnt pathway regulates myofibroblast activity in various tissues. In a sustained high Wnt activity model, myofibroblasts’ persistent activation and proliferation lead to severe tissue fibrosis (83–89). M2 macrophages play a critical role in promoting fibrotic processes across various tissues. They actively secrete a range of pro-fibrotic factors, including TGF-β1, Fibroblast growth factor 2(FGF2), PDGF, and galectin 3. These factors serve to stimulate myofibroblast proliferation, enhance their survival, activate them, and lead to the overproduction of ECM components. Additionally, macrophages produce cytokines such as IL-1, MMP-9, angiotensin (Ang)-II, and IGF-1, which trigger processes like epithelial-mesenchymal transition (EMT) and endothelial-mesenchymal transition (EndoMT) in various cell types, including tubular epithelial cells, endothelial cells, pericytes, local fibroblasts, and mesangial cells. This, in turn, leads to the transdifferentiation or activation of myofibroblasts (90, 91). Furthermore, recent research suggests that monocytes/macrophages can differentiate into collagen-producing fibroblasts or directly into myofibroblasts (92). Activated macrophages can also disrupt glomerular and peritubular capillaries, promoting hypoxia-driven fibrosis (90).

Classical TGF-β signaling via TGF-βR1 and TGF-βR2 operates the complexes containing Smad2, Smad3, and Smad4. Smad3 deficiency in mice leads to a significant decrease in myofibroblast accumulation in the kidney. It protects against renal fibrosis in different disease models, indicating a crucial role for Smad3 in the macrophage-myofibroblast transition (MMT) process. Conversely, Th2 cytokines, such as IL-4 and IL-13, can promote fibrosis in various organ-based diseases by inducing macrophage M2 polarization through the JAK-STAT pathway. In the kidney, CD4^+^ T cells promote kidney fibrosis by producing high levels of IL-4 and IL-13 and exhibiting a Th2 phenotype. Mice lacking the IL-4 receptor α-chain are protected from kidney fibrosis induced by unilateral ureteral obstruction(UUO) and folic acid, with reduced STAT6 signaling in the kidney and lower numbers of both CD206^+^ M2 macrophages and CD206^+^PDGFRβ^+^ bone marrow-derived fibroblasts (52).

In the context of studying MMT in human diseases, researchers commonly identify intermediate cells exhibiting both macrophage markers, such as CD68, and myofibroblast markers, like α-smooth muscle actin (αSMA). In a biopsy-based investigation involving patients with various native kidney diseases, researchers observed the presence of CD68^+^αSMA^+^ cells exclusively in patients with active fibrotic lesions. These dual-marker cells were not found in samples characterized by acute inflammation without fibrosis or in tissues with inactive fibrosis. Furthermore, there was a positive correlation between the number of CD68^+^αSMA^+^ cells and the total count of myofibroblasts in tissues exhibiting active fibrosis. Notably, the majority of CD68^+^αSMA^+^ cells co-expressed CD206, suggesting that macrophages undergoing MMT were predominantly of the M2 subtype (93).

In addition, injecting activated DCs into the injured heart can improve myocardial fibrosis, remodeling, and cardiac function, and this is believed to occur through the modulation of Tregs and the shift of macrophage polarization toward the M2 phenotype (94).

### 3.4 The resolution of tissue fibrosis

The formation and degradation of ECM counterbalance each other, and tissue fibrosis can be reversible whenever ECM breakdown predominates. The outcome of wound healing is determined by a delicate equilibrium between pro-fibrotic and anti-fibrotic factors (95). Macrophages secrete MMPs, a family of proteases involved in the degradation of different types of ECM proteins, e.g., during the resolution of fibrosis (96). In cases of liver injury, myofibroblasts and activated hepatic stellate cells produce tissue inhibitors of MMPs (TIMPs), inhibiting macrophage-secreted MMPs’ activity. This inhibition contributes to the progressive deposition of ECM and the accumulation of scar tissue in the liver (97, 98). Following the cessation of the injury stimulus, macrophages undergo a phenotype transition towards an anti-fibrotic phenotype (99, 100). Apart from clearing cellular debris, such macrophages secrete several fibrinolytic MMPs, such as MMP9, MMP12, and MMP13, which facilitate the degradation of fibrotic ECM (101–103). In addition, macrophages also can generate additional mediators that offer protection against kidney fibrosis. These include collagenases, nitric oxide (NO), and bone morphogenic protein-7 (BMP-7) (104).

Due to recent technical advances, single-cell RNA sequencing (scRNA-seq) discovered macrophage populations with abundant heterogeneity, functionality, and subtle differences in their *in vivo* phenotypes. Sommerfeld et al. employed a platform that combined single-cell technology and functional assessment to elucidate how macrophages reacted to diverse microenvironments and demonstrated that controlling M1/M2 macrophage populations can determine the outcome of tissues in fibrosis (105).

## 4 Summary, knowledge gaps, and research opportunities

Focal fibrosis is an essential element in tissue repair and stabilizes the surrounding parenchyma. The presence of fibrotic tissue during the healing process can be transient or accumulate over an extended period. The duration of fibrosis ultimately determines the formation of scar tissue or parenchymal reconstitution. The degree and duration of injury, the body response to invading microorganisms, and changes in inflammation over time modulate the outcome.

Over the years, studies revealed that the MP system is involved in all phases of this process. During homeostasis, circulating monocytes migrate into tissues to become tissue-resident macrophages or DCs. Upon tissue injury, the surviving differentiated parenchymal cells can enhance their functional capacity by undergoing hypertrophy through polyploidization, and the changing tissue environment primes different mononuclear phagocyte phenotypes and determines their function in changing spatial and temporal contexts. During injury-related necroinflammation, macrophages polarize to the M1 type, performing host defense and pro-inflammatory functions, and secrete pro-inflammatory cytokines that support Th1 cells to function. cDC2s contribute to tissue inflammation in a complementary manner, activate and release cytokines after recognizing immunogenic substances, promote the recruitment of neutrophils, and function as innate immune response inducers. During the inflammation subsidence period, macrophages polarize to the M2 type, which mainly plays an anti-inflammatory function, produces anti-inflammatory mediators, promotes angiogenesis, and secrete growth factors beneficial to tissue healing. In contrast, cDC1s regulate inflammatory processes by sustaining Tregs. Furthermore, cDC1s can limit the activity of cytotoxic T cells through the signal from PD-L1. In the post-injury repair and fibrosis phase, M2 macrophages generate substantial quantities of pro-fibrotic factors that enhance myofibroblasts’ proliferation, survival, and activation. Conversely, macrophages play a crucial role in promoting ECM degradation during the final stage of fibrotic regression by secreting MMPs.

In recent years, scRNAseq has enabled the characterization of the functions of macrophages, particularly in various microenvironments. The tissue fate during inflammation and fibrosis might be determined by manipulating functional changes of these cells, which is a potential and promising therapeutic target for post-injury repair. For example, various cells of the MP lineage express macrophage colony-stimulating factor receptors (M-CSFR), including tumor-associated macrophages. As a result, chimeric antigen receptor T-cell immunotherapy (CAR-T) targeting M-CSFR has been used in tumor treatment (106). Besides, the therapeutic effect also can be forecasted by scRNAseq (107). However, the relationship between MP and fibrosis presents numerous unsolved questions and challenges, offering valuable research opportunities for further investigation and understanding (**Table 3**).

**Table 3:** Research opportunities for further investigation and understanding.

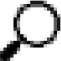 The single-cell analysis offers new research opportunities.
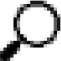 The functional roles of different MP lineages remain to be dissected, which requires lineage-specific tools, such as the Cre-lox system.
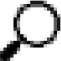 The roles of MP in tissue polyploidization and cell hypertrophy remain largely unknown. As compensatory hypertrophy is an essential adaption mechanism, MP likely support this process by secreting specific ligands or modulators.
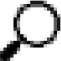 Whether specifically reducing tissue fibrosis improves organ function is unclear and may differ in various organs. While anti-fibrotic therapy appears to benefit lung function, there is a lack of evidence supporting its effectiveness in treating liver, kidney, and heart fibrosis. As the enthusiasm for targeting fibrosis as a potential treatment continues, there is a growing need for further interventional evidence to understand its therapeutic potential better.
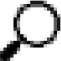 Whether manipulating MP is a way to improve tissue regeneration after injury needs more exploration. We show that MP can benefit tissue responses in multiple directions, suggesting that targeting MP to enhance tissue recovery could be a viable option.

MP (Mononuclear Phagocytes).

## Author contributions

H-JA generated the concept. HL wrote the first draft. The manuscript was further modified and edited by all authors. All authors contributed to the article and approved the submitted version. 
